# Supplementary material for: Stability and variation of brain-behavior correlation patterns across measures of social support
Source: Imaging Neurosci (Camb). 2024 Apr 18;2:imag-2-00133. doi: 10.1162/imag_a_00133 (PMC12247621; doi:10.1162/imag_a_00133)
Supplement: Supplementary Material [file imag_a_00133-supp.pdf]

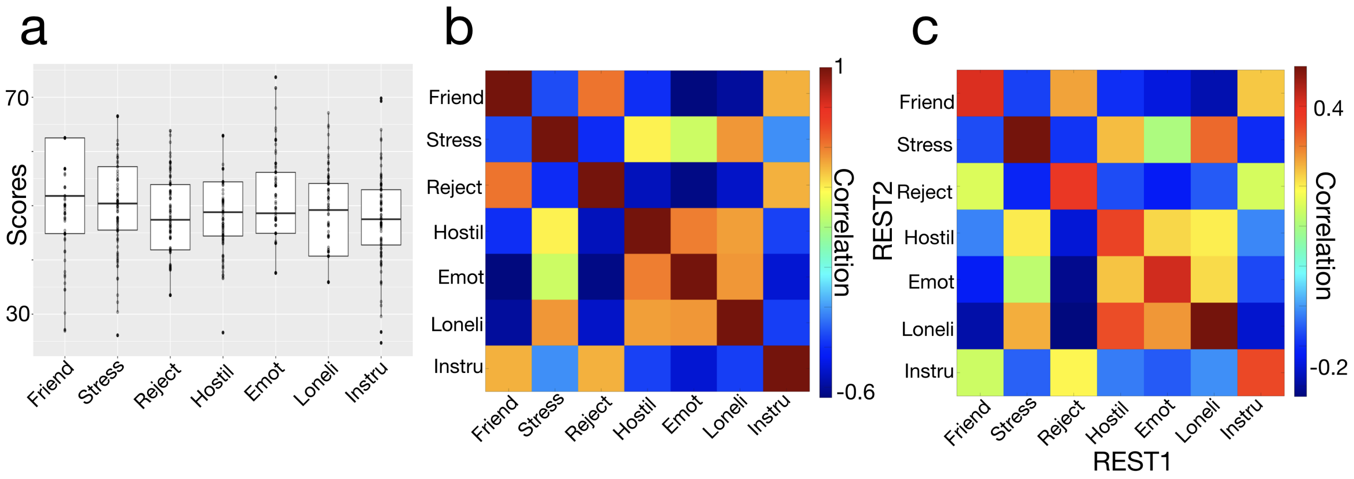

Figure 1: **Social support measures.** Panel (a) shows the distributions of scores for each of the social support measures. Panel (b) shows the correlations between all of the measures. Panel (c) shows the correlations across rest scans between each measure and the functional connectivity edge weights.

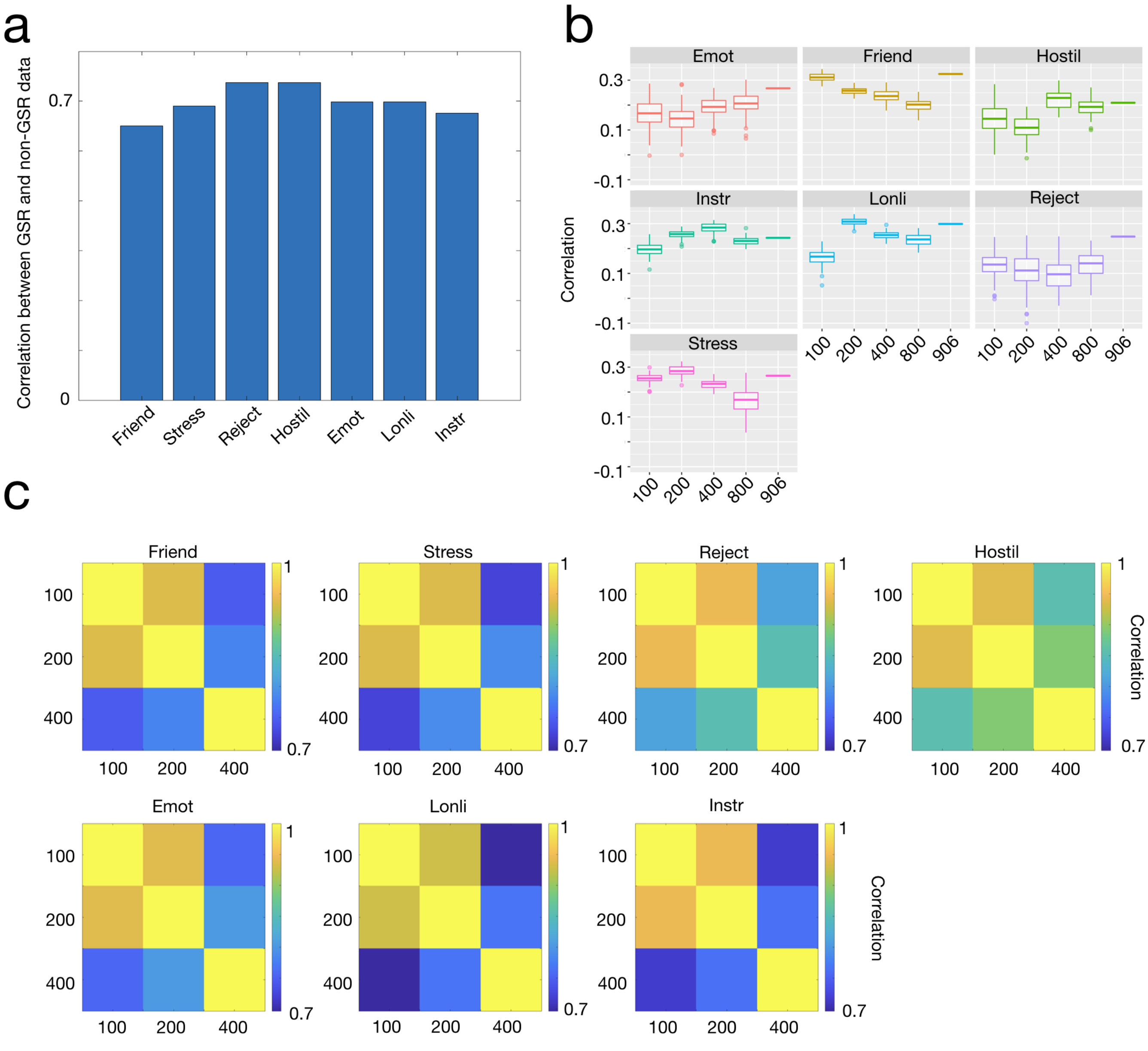

Figure 2: **Generalizability of results.** Panel (a) shows the correlations between data with and without global signal regression. For each measure, we correlate the matrices of correlations between edge weights and the social support measure. Correlations range from 0.551 (for the Friendship measure) to 0.6375 (for the Hostility measure). We also compared our original data to samples of various sizes from the HCP dataset (b). For each sample size (n = 100, 200, 400, 800), we subsampled n individuals at random from the entire HCP dataset. For each subsample, we computed the correlation between the original edge weight-social support correlation matrix and the subsampled edge weight-social support correlation matrix. Additionally, for all HCP subjects that had complete data (n = 906), we computed the correlation with the original data. All correlations are shown by measure in the box plots in panel (b). In panel (c), we show the correlations between the edge weight-social support correlation matrices across different parcellations. While the 100 node and 200 node parcellations tend to be more similar to each other than either is to the 400 node parcellation, all correlations are greater than 0.6968 (observed for the Loneliness measure).

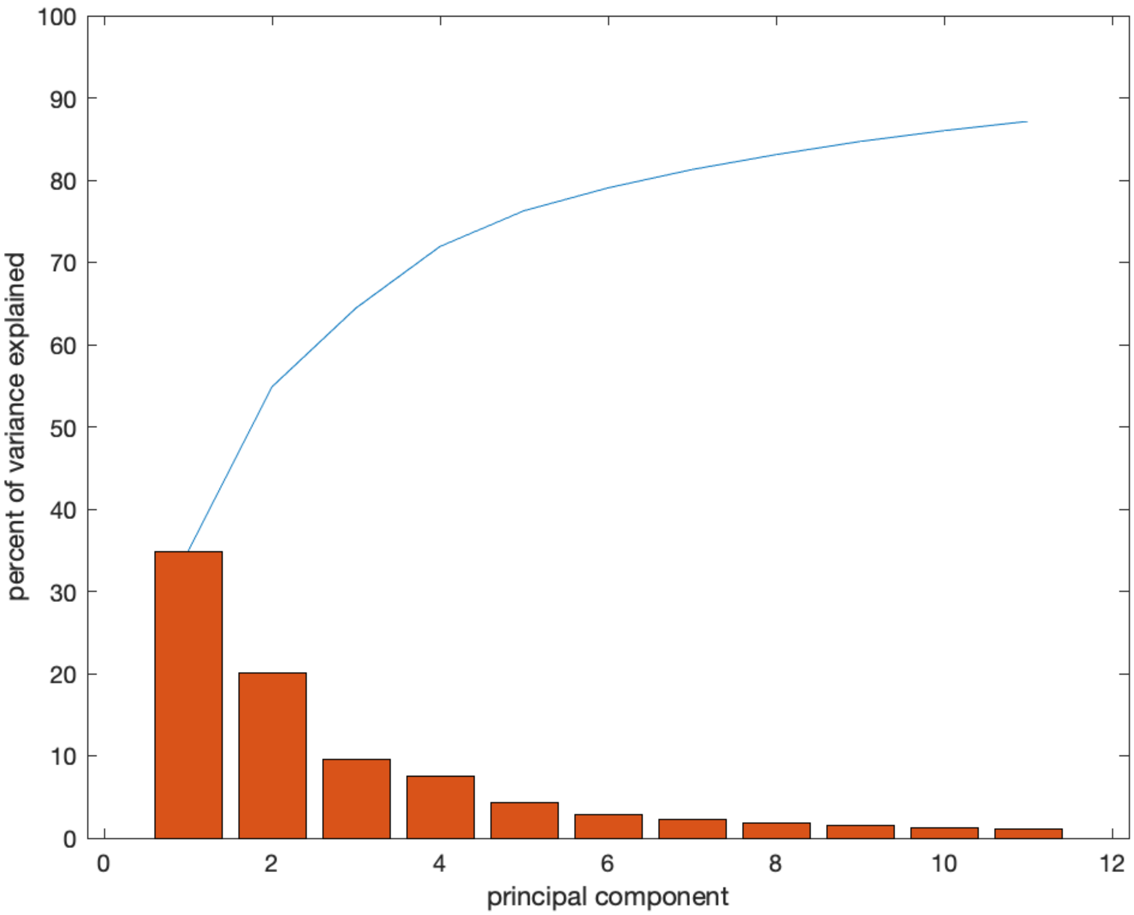

Figure 3: **Cumulative variance explained.** This figure shows the cumulative variance explained by the top 11 components that explain at least 1% of the total variance of parameter space each. Principal component 1 explains about 35% of the variance, principal component 2 explains just over 20% of the variance, and principal component 3 explains about 9.5%.

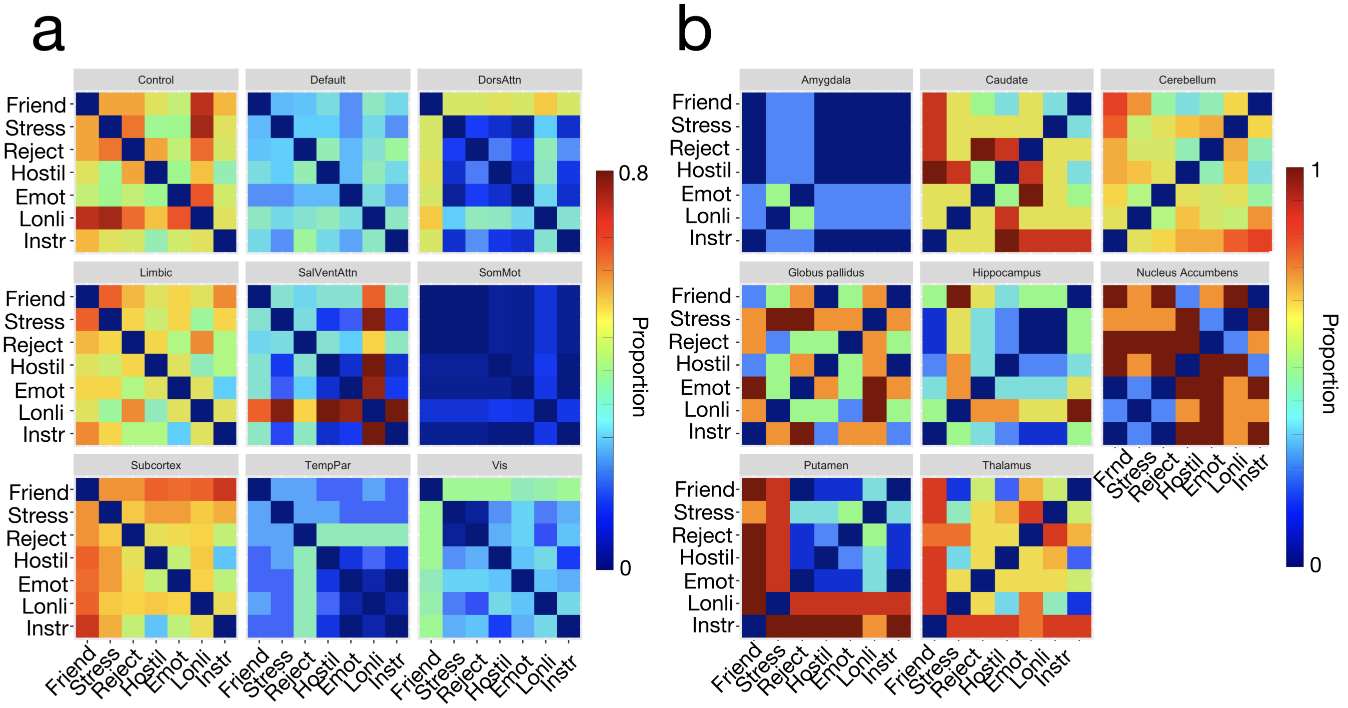

Figure 4: **Breakdown of community switches between layers by system.** In Figure 5 in the main text, we show the global pattern of the proportion of nodes that switch their community affiliation between each pair of layers. Here we break down for different canonical systems in panel (a) and for different subcortical nuclei in panel (b).

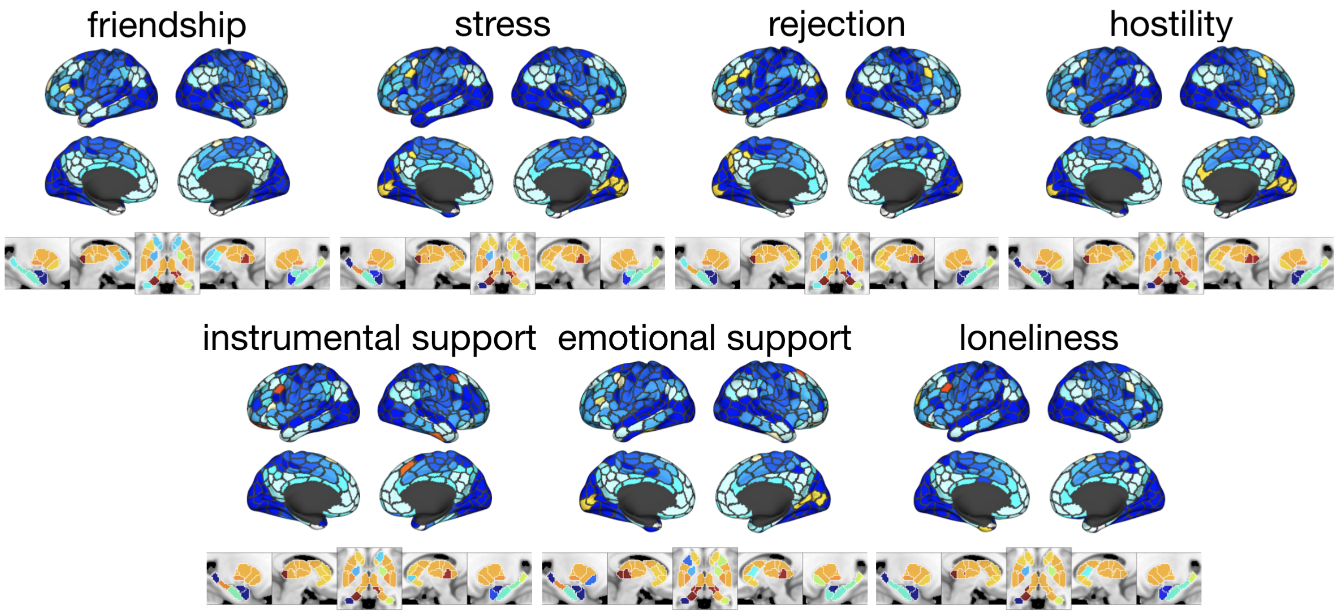

Figure 5: **Communities for principal component 2.** Community structure across all social support measures at the point in parameter space whose coefficient for principal component 2 has the largest magnitude ($\gamma = 0.35, \omega = 0.01$). There are 25 communities total, all of which are present in each layer. The MNI coordinates for the five panels of subcortical communities are, from left to right: x = -23, x = 10, z = -3, x = 13, x = 27.

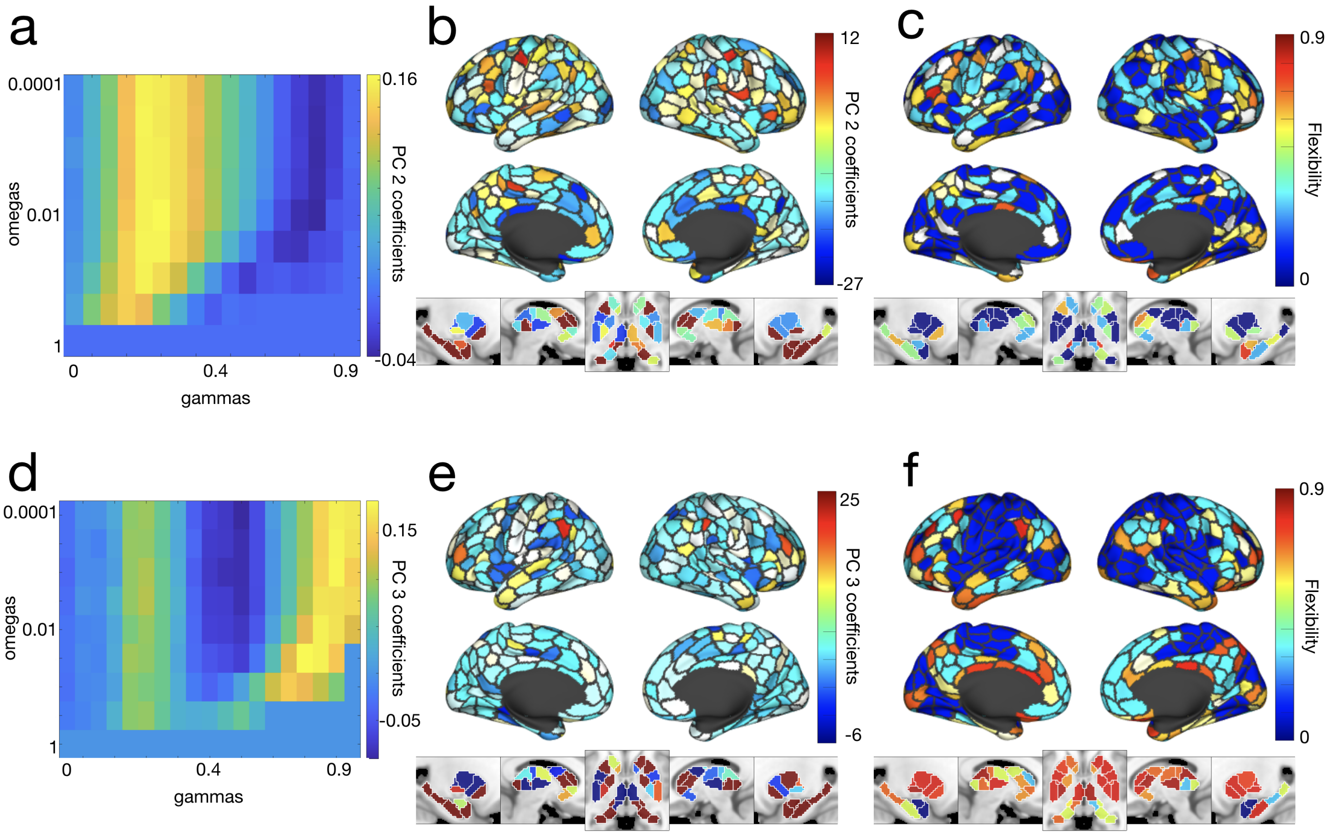

Figure 6: **Principal components 2 and 3.** The top row presents data from the second principal component, while the bottom row presents data from the third. Panels (a) and (d) show the PC coefficients for each point in parameter space. Panels (b) and (e) show the PC components projected into brain space. Panels (c) and (f) show the flexibility of cortical and subcortical nodes at the points in parameter space for which the magnitude of the coefficient of the PC is maximized. The MNI coordinates for the five panels of subcortical PC components and flexibility are, from left to right: x = -23, x = 10, z = -3, x = 13, x = 27.

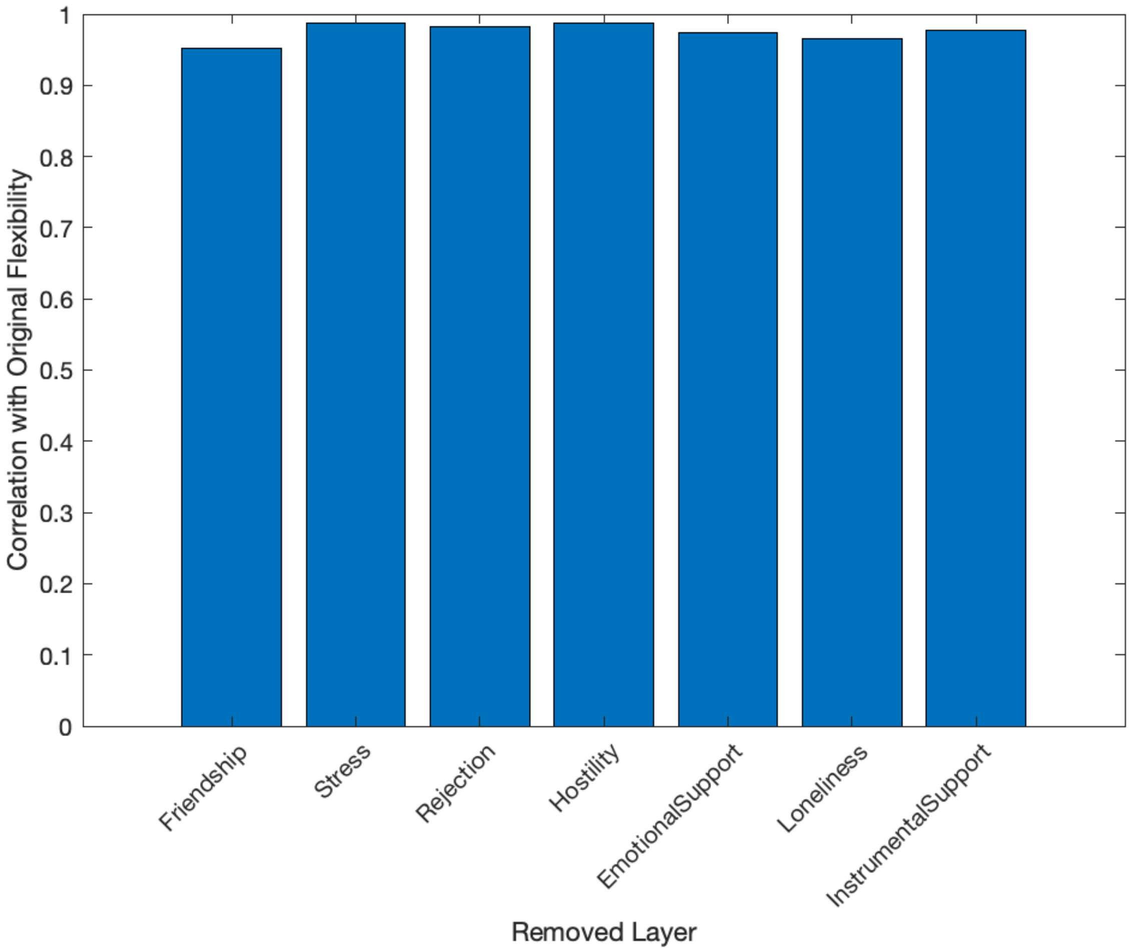

Figure 7: **Correlation with Original Flexibility.** We recalculated flexibility by systematically excluding each layer in turn then calculating the correlation between recalculated and original flexibility values. While *Flexibility*, when excluded, had the lowest correlation, all correlations were $\rho \geq 0.95$ (and all ps ≤ 0.001).

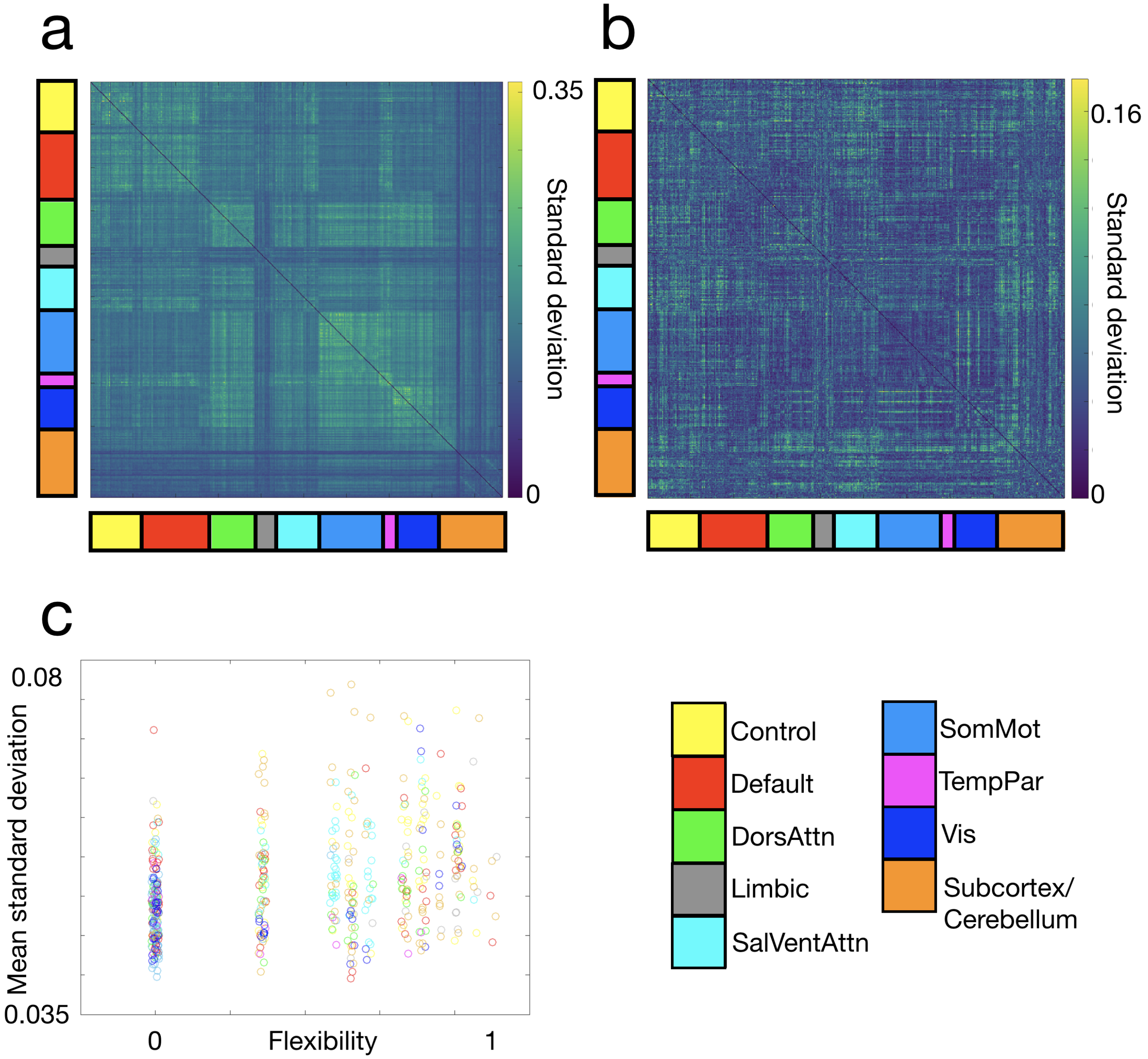

Figure 8: **Variability of correlations.** Panel (a) shows that variability of functional connectivity edge weights across individuals. Panel (b) shows the variability of edge weight - social support correlations across measures. Panel (c) shows nodal flexibility plotted against the mean standard deviation of edge weight - social support correlations (the mean of a row of the matrix in panel (b)). There is a weak correlation between flexibility and mean standard deviation ($\rho = 0.337, p < 0.001$). Colors indicate canonical communities.
